# Differences and Similarities between the Lung Transcriptomic Profiles of COVID-19, COPD, and IPF Patients: A Meta-Analysis Study of Pathophysiological Signaling Pathways

**DOI:** 10.3390/life12060887

**Published:** 2022-06-14

**Authors:** Daniel Aguilar, Adelaida Bosacoma, Isabel Blanco, Olga Tura-Ceide, Anna Serrano-Mollar, Joan Albert Barberà, Victor Ivo Peinado

**Affiliations:** 1 Biomedical Research Networking Center in Hepatic and Digestive Diseases (CIBEREDH), 28005 Madrid, Spain; daguilar@daguilar.net; 2 Institut d’Investigacions Biomèdiques August Pi i Sunyer (IDIBAPS), 08036 Barcelona, Spain; adelaidabosacoma@gmail.com (A.B.); iblanco2@clinic.cat (I.B.); olgaturac@gmail.com (O.T.-C.); anna.serranomollar@iibb.csic.es (A.S.-M.); jbarbera@clinic.cat (J.A.B.); 3 Biomedical Research Networking Center in Respiratory Diseases (CIBERES), 28029 Madrid, Spain; 4 Department of Pulmonary Medicine, Hospital Clínic, University of Barcelona, 08007 Barcelona, Spain; 5 Girona Biomedical Research Institute (IDIBGI), 17190 Girona, Spain; 6 Department of Experimental Pathology, Institut d’Investigacions Biomèdiques de Barcelona (IIBB), CSIC-IDIBAPS, 08036 Barcelona, Spain

**Keywords:** chronic lung disease, transcriptome, interactome, cluster analysis, bioinformatic analysis, molecular pathway, inflammation

## Abstract

Coronavirus disease 2019 (COVID-19) is a pandemic respiratory disease associated with high morbidity and mortality. Although many patients recover, long-term sequelae after infection have become increasingly recognized and concerning. Among other sequelae, the available data indicate that many patients who recover from COVID-19 could develop fibrotic abnormalities over time. To understand the basic pathophysiology underlying the development of long-term pulmonary fibrosis in COVID-19, as well as the higher mortality rates in patients with pre-existing lung diseases, we compared the transcriptomic fingerprints among patients with COVID-19, idiopathic pulmonary fibrosis (IPF), and chronic obstructive pulmonary disease (COPD) using interactomic analysis. Patients who died of COVID-19 shared some of the molecular biological processes triggered in patients with IPF, such as those related to immune response, airway remodeling, and wound healing, which could explain the radiological images seen in some patients after discharge. However, other aspects of this transcriptomic profile did not resemble the profile associated with irreversible fibrotic processes in IPF. Our mathematical approach instead showed that the molecular processes that were altered in COVID-19 patients more closely resembled those observed in COPD. These data indicate that patients with COPD, who have overcome COVID-19, might experience a faster decline in lung function that will undoubtedly affect global health.

## 1. Introduction

COVID-19 is a pandemic respiratory disease, caused by the severe acute respiratory syndrome coronavirus 2 (SARS-CoV-2), which is associated with considerable morbidity and mortality. Most patients improve over time but a significant number have lung function and radiological alterations a year after discharge [1]. 

To date, available data indicate that more than a third of recovered COVID-19 patients will develop fibrotic-like abnormalities [2,3,4]. Furthermore, 47% have an altered diffusing capacity of the lungs for carbon monoxide (DLCO) and 25% have a reduced total lung capacity [2]. There are serious doubts whether the observed fibrotic abnormalities resolve fully, and indeed, there is evidence that a non-negligible percentage will develop irreversible pulmonary fibrosis [5]. In a 15-year follow-up study of 71 survivors from SARS-CoV-1 infection, the coronavirus that emerged in Southeast Asia in 2003, pulmonary diffusion abnormalities were observed in approximately one-third of patients [6], and a 6-month follow-up study of computed tomography images for 40 SARS-CoV-1 patients revealed long-term sequelae, such as air trapping, ground-glass opacities, reticulations, and bronchial distortion, in more than half of the participants [7]. One-year follow-up studies in COVID-19 patients showed that although the proportion of radiological abnormalities in the lung is very significant at 3 months after hospital discharge, similar to those observed in SARS-CoV-1, radiological changes only persisted one year later in about 5–20% of them [8,9,10]. Nonetheless, given the large number of people affected by COVID-19, there is a real risk of a significant number of patients experiencing lung function issues in the long term.

Early identification of the subpopulation that could develop pulmonary fibrosis is of great importance if one wants to delay or diminish the development of lung injury [11]. Furthermore, even a relatively small degree of residual but non-progressive fibrosis could result in considerable morbidity and mortality in older patients who have had COVID-19, especially if they have pre-existing pulmonary conditions. The mechanisms by which SARS-CoV-2 causes lung damage and/or induces fibrotic lesions are very poorly understood. Potential triggers are the cytokine storm induced by the viral antigen and/or the high airway pressure and hyperoxia during mechanical ventilation. If we could relate the activation of molecular mechanisms with the development of pulmonary fibrosis, we could perhaps have an opportunity to stop, or even reverse, the long-term fibrotic processes in patients with COVID-19.

Using mathematical interactome-based cluster analysis, this study aimed to compare the most important activated molecular pathways in the lungs of patients who died of COVID-19, using the transcriptomic fingerprints of patients with irreversible pulmonary fibrosis, as occurs in idiopathic pulmonary fibrosis (IPF). We also compared the molecular pathways triggered in COVID-19 with those triggered in COPD, because although IPF and COPD have different etiologies, both are chronic inflammatory diseases with a fibrotic component [12]. Expanding on this topic, diagnoses of IPF or COPD represent risk factors in COVID-19 [13,14], which may be because of synergism that aggravates the disease. Therefore, comparison among the three diseases may help us to understand the pathophysiology of COVID-19. Understanding the common molecular pathways will facilitate better stratification of pulmonary risk during SARS-CoV-2 infection, will help to implement preventive strategies, and will facilitate the longitudinal monitoring of pulmonary responses to specific treatments, especially in patients with pre-existing chronic lung diseases.

## 2. Methods

### 2.1. Expression Datasets

We analyzed data from three independent expression datasets: an IPF dataset, a COVID-19 dataset, and a COPD dataset. To ensure that the three datasets were fully comparable, we restricted genes to only those present in the three experiments and transformed all unofficial gene names to approved gene names, when possible, in accordance with the HUGO Gene Nomenclature Committee (HGNC) resource (provided this did not generate duplications or ambiguities). 

#### 2.1.1. COVID-19 Dataset

We used a study of biopsy samples from SARS-CoV-2-infected patients by Desai et al. [15], which used RNA-seq, to form the COVID-19 dataset (https://www.ncbi.nlm.nih.gov/geo/query/acc.cgi?acc=GSE150316, accessed on 20 July 2020). This included 24 lung biopsies from infected patients (age, 32–89 years). Although these patients had pathologies prior to infection, none of these patients had a diagnosis of COPD or IPF. Raw counts were transformed to logCPM values and normalized with the *voom* tool in the limma package. Rows with zero counts for all columns were removed. Probes lacking a HGNC gene name were discarded. Analysis of differential gene expression was performed using the lmFit function in the limma package, and we considered a more relaxed differential expression cut-off (|FC| > 1.5; *p* < 0.05) than for the IPF dataset. This was necessary because we expected a sizable fraction of differentially expressed genes (DEGs) to be lost due to interactome incompleteness and because we considered only those edges connecting two DEGs.

#### 2.1.2. IPF Dataset

We used data on IPF lung biopsies from the study by De Pianto et al. [16], which used gene expression microarrays, to form the IPF dataset (https://www.ebi.ac.uk/arrayexpress/experiments/E-GEOD-53845/, accessed on 9 March 2020). This included 40 patients and 8 controls (age, 18–80 years). Raw data from microarray files were extracted, background corrected, and normalized with the voom tool in the limma R package. Values for within-array replicated probes were replaced with their average and probes without a HGNC gene name were discarded. Differential expression analysis was carried out with the lmFit function from the limma package. Genes were differentially expressed if |FC| > 2 and FDR-adjusted *p* < 0.05.

#### 2.1.3. COPD Dataset

We used a study of respiratory tract samples of patients with and without COPD by van Dyck et al. [17], which used gene expression microarrays, to form the COPD dataset (https://www.ebi.ac.uk/arrayexpress/experiments/E-MTAB-1690/, accessed on 2 April 2020). The data included 21 patients with COPD and 14 healthy non-smoker controls. Patients with head, neck, or lung cancer were excluded. Data from raw microarray files were extracted, background corrected, and normalized with the voom tool in the limma R package. Values for within-array replicated probes were replaced by averages. Probes without a HGNC gene name were discarded. Differential expression analysis was carried out with the lmFit function in the limma package, and genes were considered differentially expressed with an |FC| > 2 and an FDR-adjusted *p* < 0.05.

### 2.2. Interactome Analysis

#### 2.2.1. Data Sources

The human interactome contains information about functional connections between genes (e.g., physical interactions between gene products, transcriptional regulation, metabolic associations, etc.) mined from the literature. The study of the interactome has contributed to the identification of disease mechanisms and disease-associated genes [18,19]. The human interactome was built by combining two data sources: (1) data derived from a Reactome database [version 022717] [19], removing any interactions labeled as predicted, and (2) data derived from STRING database [version 11] [20], only considering interactions with a score >700 (high-confidence interactions). The combined interactome had 17,517 nodes (genes) and 471,401 edges (connections).

#### 2.2.2. COVID-19, IPF, and COPD Interactomes

We built the interactome of each dataset by mapping DEGs onto the interactome, while keeping only those edges connecting two DEGs. We next performed a clustering of the interactome which identified densely interconnected groups of genes (named clusters). Clusters usually contain functionally related genes, given that a high level of interconnectedness implies functional association between them [21,22]. Clustering was performed with the Markov cluster algorithm, using 2 as an inflation parameter [23]. Only clusters with ≥10 genes were considered. For the COVID-19 dataset, we removed clusters that did not contain at least one differentially expressed gene.

### 2.3. Functional Characterization

The sources of functional annotation were the *biological process* and *molecular function* ontologies of the Gene Ontology (GO) database [The Gene Ontology Consortium, 2019] [24]. Only annotations with experimental evidence codes were considered. Functional characterization of clusters was performed using the BinoX software [25]. BinoX exploits the information within the human interactome to evaluate the functional interactions between a set of genes and a set of genes annotated with a particular function. This allows for the identification of functional relationships that would go unnoticed with traditional association tests (e.g., a Fisher’s exact test) [26,27]. Enrichment in GO terms was considered significant with an FDR-adjusted *p* < 0.05. To facilitate visualization of the results, GO terms were classified into 32 broad functional categories (listed in Table 1).

### 2.4. Functional Connectivity

For each disease, we calculated the functional connectivity value between clusters. This allowed us to measure the functional similarity between clusters, where a high connectivity implies high functional association between them (i.e., their gene sets contribute to the same biological processes) [20,26]. After calculating the functional connectivity between clusters, the global functional distance between diseases was calculated as the maximal functional connectivity value between any cluster of one disease to any cluster of the other. 

Connectivity between any two clusters, *i* and *j,* was measured in a three-step process. (1) Firstly, we calculated the fraction of genes common to both clusters as:

(1) $$n_{\mathrm{common}}{=n}_{\mathrm{ij}}{+n}_{\mathrm{ji}}$$

where n_ij_ is the number of genes of cluster *i* present in cluster *j*, and n_ji_ is the number of genes of cluster *j* present in cluster *i*. (2) For the genes unique to each cluster, we used the interactomic data to check whether they were connected to genes in the other cluster. We then calculated the overall connectivity as:

(2) $$n_{\mathrm{connected}}{=e}_{\mathrm{ij}}{+e}_{\mathrm{ji}}$$

where e_ij_ is the number of genes of cluster *i* connected to any gene in cluster *j*, and e_ji_ is the number of genes of cluster *j* connected to any gene in cluster *i*. (3) Lastly, the connectivity between the two clusters was calculated as:

(3) $$\mathrm{connectivity}=\frac{n_{\mathrm{common}}{+n}_{\mathrm{connected}}}{N_{i}{+N}_{j}}$$

where *N_i_* is the number of genes in cluster *i*, and *N_j_* is the number of genes present in cluster *j*. Functional connectivity value will range from 0 (neither shared genes nor connections between genes in the clusters) to 1 (all genes in one cluster are either present or connected to at least one gene in the other cluster). This value can be interpreted as functional relatedness between groups of genes [27,28,29].

Given that any pair of clusters has a functional connectivity value (both within and between diseases), clusters could then be clustered according to their connectivity. This would identify groups of clusters (i.e., clusters of clusters) that are functionally related. We used the hclust function in R with a single-linkage method, and the optimal partition was measured by Pearson’s gamma method, using the cluster.stats function of the fpc R package (version 2.2-9) [29]. Dendrograms were plotted with the dendextend package in R [30].

The global functional distance between two diseases was calculated as the maximal functional connectivity value between any cluster of one disease to any cluster on the other.

## 3. Results

### 3.1. Data Analysis

**Figure 1:**
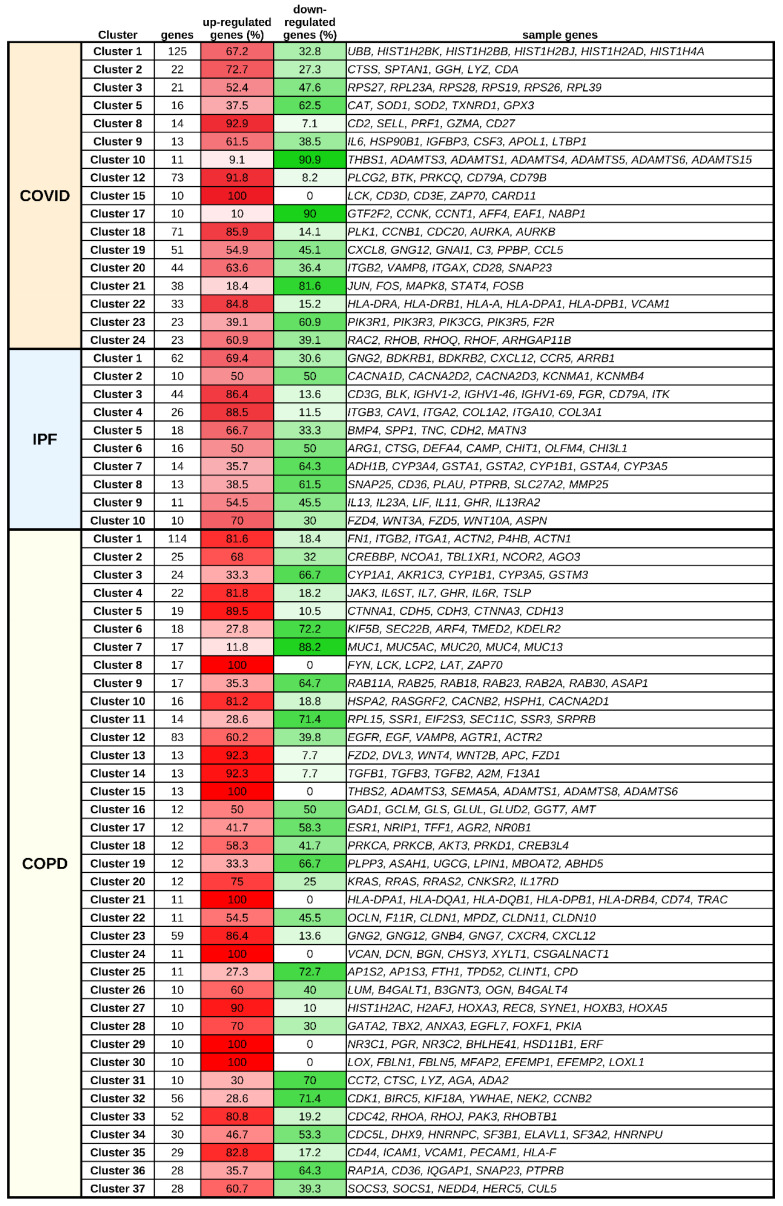
**Summary of clusters obtained in COVID-19, IPF, and COPD.** The percentages of upregulated and downregulated genes in each cluster are shown in gradients of red and green, respectively.

Figure 1 summarizes the differential expression results for the study datasets, with the complete clusters provided in Supplementary Materials, Figures S1–S3 for the IPF, COPD, and COVID-19 datasets, respectively.

### 3.2. Interactome Analysis

**Figure 2:**
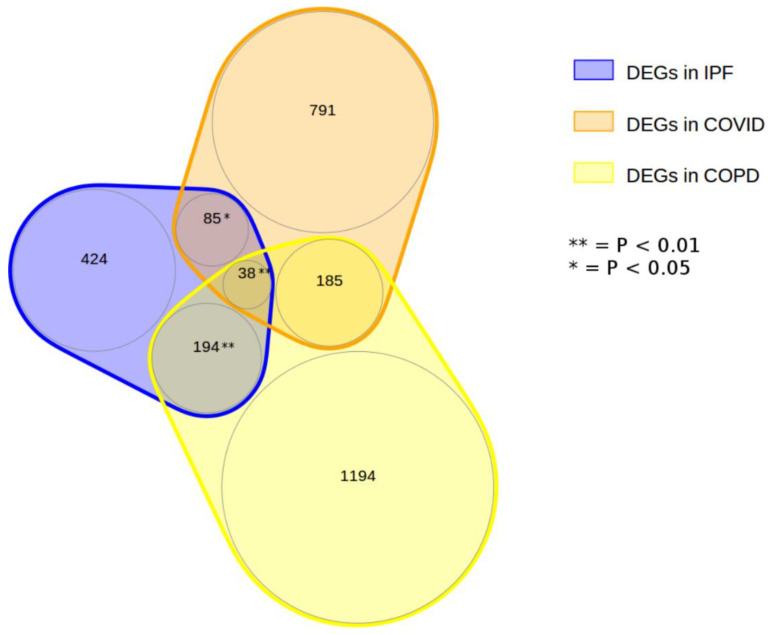
Representation of the number of differentially expressed genes (DEGs) for COVID-19, IPF, and COPD. As shown, 123 (38 + 85) IPF genes (16.6%) and 223 (38 + 185) COPD genes (13.8%) are shared with COVID-19.

We mapped the DEGs onto the interactome to exploit that information to characterize them by function. The number of DEGs from the COVID-19 dataset present in the interactome was 1678 (49.3% of all COVID-19 DEGs), and for the IPF dataset, this number was 872 (75.8% of all IPF DEGs). Most DEGs not present in the interactome were either non-protein-coding genes or genes without official names that did not contribute to the functional characterization of the clusters (82.84% in the IPF dataset, 91.4% in the COVID-19 dataset, and 66.93% in the COPD dataset). Figure 2 shows the overlap of DEGs for COVID-19, IPF, and COPD. As shown, several IPF genes (*n* = 85; 11.5% of all) or COPD genes (*n* = 185; 11.5% of all) were shared exclusively with COVID-19 DEGs. Interactome mapping and clustering found 24 clusters for COVID-19 DEGs, 11 clusters for IPF DEGs, and 37 clusters for COPD DEGs (Supplementary Figures S1–S3).

### 3.3. Functional Characterization

**Figure 3:**
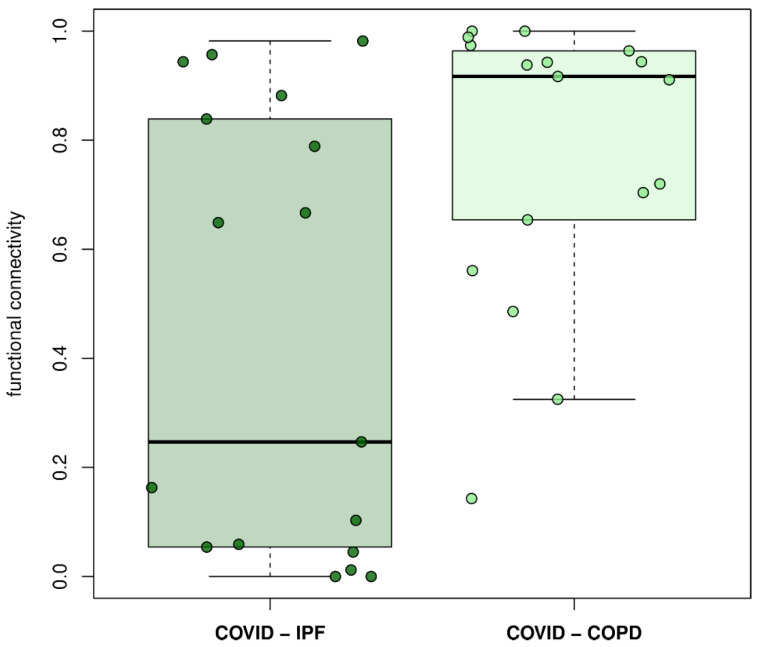
**Global functional distance between COVID-19 and IPF/COPD**. Difference is significant (*p* = 0.0051; Wilcoxon–Mann–Whitney test).

**Figure 4:**
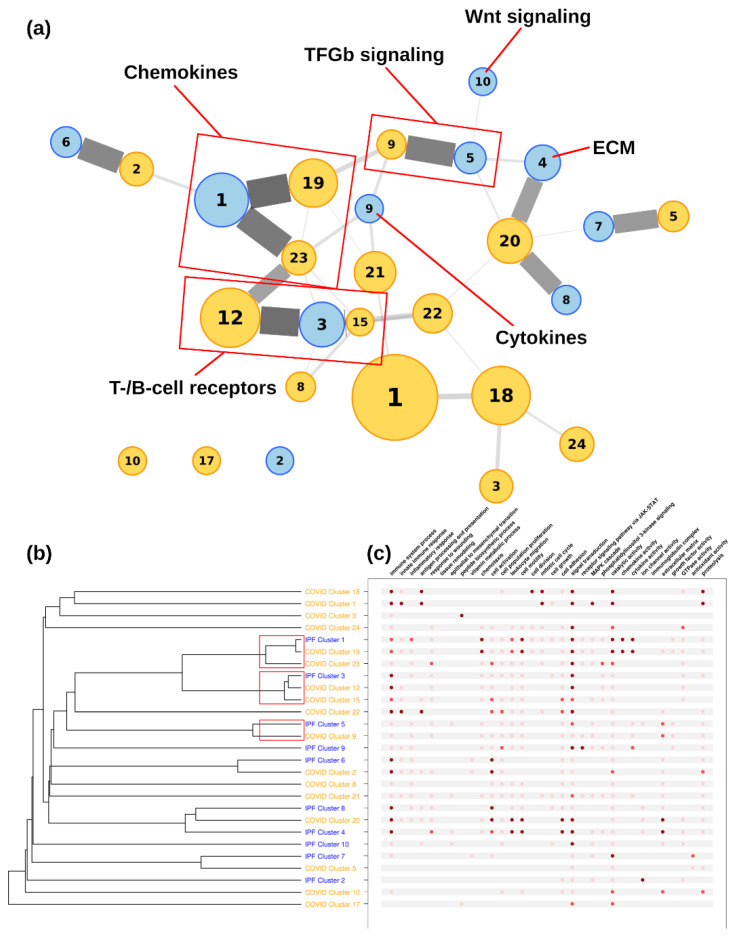
**Functional connectivity between COVID-19 and IPF clusters.** COVID-19 clusters are shown in orange, IPF clusters are shown in blue. (**a**) Connectivity represented as a network of clusters. Node size is proportional to the number of genes and edge thicknesses are proportional to the functional connectivity. Red boxes indicate clusters within the optimal partition. (**b**) Connectivity represented as a dendrogram. (**c**) Main functional annotations. Dots show the significance of the functional enrichment (dark red = adjusted *p* < 5 × 10^−100^; medium red = adjusted *p* < 5 × 10^−50^; light red = adjusted *p* < 5 × 10^−5^).

**Figure 5:**
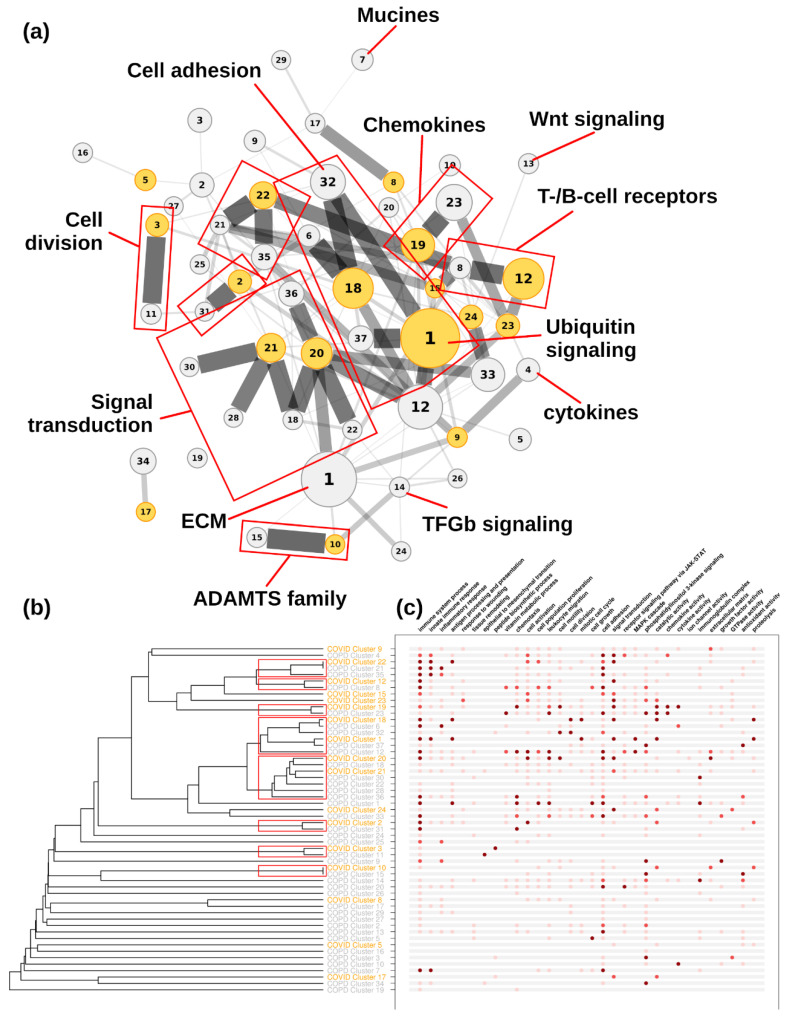
**Functional connectivity between COVID-19 and COPD clusters**. COVID-19 clusters are shown in orange, COPD clusters are shown in grey. (**a**) Connectivity represented as a network of clusters. Node size is as in Figure 4. Red boxes indicate clusters within the optimal partition. (**b**) Connectivity represented as a dendrogram. (**c**) Main functional annotations. Colors are as in Figure 4.

Functional annotation and the main functional categories of the clusters are summarized in Figure 3, Figure 4 and Figure 5 for the COVID-19, IPF, and COPD datasets, respectively.

### 3.4. Network Comparisons

**Figure 6:**
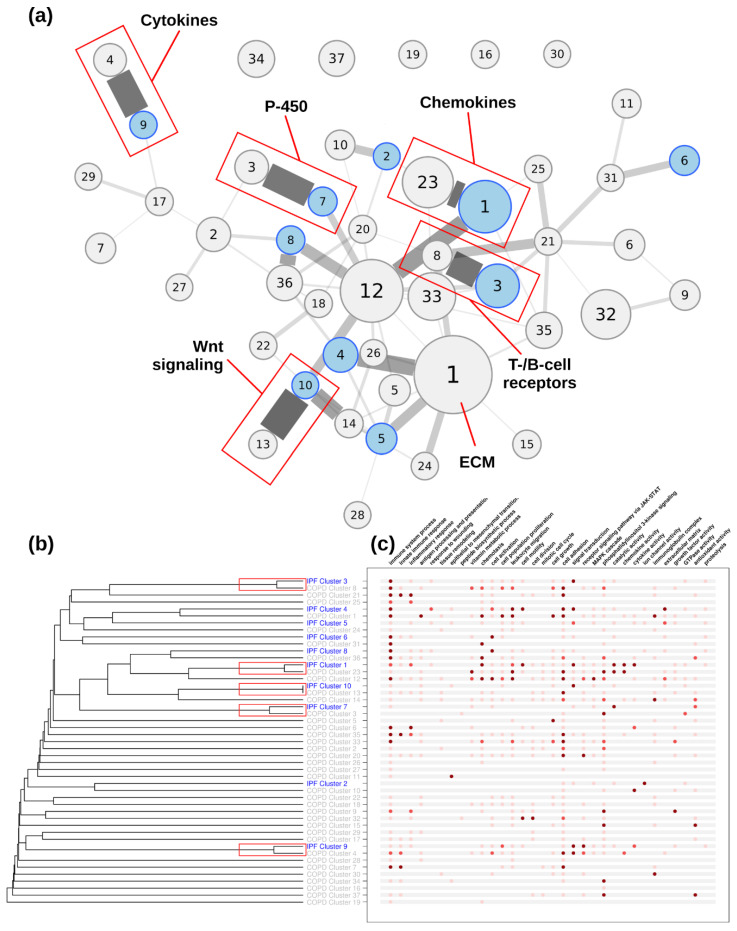
**Functional connectivity between IPF and COPD clusters.** IPF clusters are shown in blue, COPD clusters are shown in grey. (**a**) Connectivity represented as a network of clusters. Node size is as in Figure 4. Red boxes indicate clusters within the optimal partition. (**b**) Connectivity represented as a dendrogram. (**c**) Main functional annotations. Colors are as in Figure 4.

Figure 3 shows the functional similarity between COVID-19–IPF and COVID-19–COPD, revealing greater functional proximity between COVID-19 and COPD than between COVID-19 and IPF (*p* < 0.01; WMW test). Next, we measured the functional similarity between different disease clusters (COVID-19, IPF, and COPD). The two-to-two comparisons between diseases are shown in the dendrograms in Figure 4, Figure 5 and Figure 6 for COVID-19–IPF, COVID-19–COPD, and IPF–COPD, respectively.

#### 3.4.1. COVID-19–IPF Connectivity

The analysis of functional proximity between the IPF and COVID-19 clusters showed closeness of three IPF clusters (clusters 1, 3, and 5) to five clusters of COVID-19 (clusters 19, 23, 12, 15, and 9) (see red boxes in Figure 4). The highest functional similarities were between IPF cluster 1 and COVID-19 clusters 19 and 23, and between IPF cluster 3 and COVID-19 clusters 12 and 15. The first group of functionally related clusters (IPF cluster 1, COVID-19 clusters 19 and 23) corresponds to activation of the chemokine subcategory and its receptors, and is one of the most important categories (by number of genes) in both pathologies. Most genes in these clusters were upregulated. The second group of clusters (IPF cluster 3, COVID-19 clusters 12 and 15) represents a set of genes, receptors, and immunoglobulins present in cells of the immune system (T and B cells) that participate in signal transduction and dynamic regulation of the inflammatory response.

#### 3.4.2. COVID-19–COPD Connectivity

Analysis of functional proximity between COVID-19 and COPD clusters showed closeness between 10 COVID-19 clusters (1, 2, 3, 10, 12, 18, 19, 20, 21, and 22) and 16 COPD clusters (15, 11, 31, 30, 18, 12, 37, 32, 6, 23, 8, 22, 28, 36, 35 and 21) (see red boxes in Figure 5). The greatest functional similarity was present between clusters 10, 18, and 22 of COVID-19 with clusters 15, 6, and 21 of COPD, respectively. COVID-19 clusters 2, 12, and 19 also maintained functional similarity with IPF and COPD. Genes belonging to clusters 15, 6, and 21 in COPD participate in processes related to the immune response, but several genes of COPD cluster 15 (COVID-19 cluster 10) are related to the thrombospondin and metalloproteinase family, which have roles in connective tissue regulation, angiogenesis, and mesenchymal cell migration. However, the genes upregulated in COPD were mostly downregulated in COVID-19, indicating a functional divergence. The genes of COPD cluster 21 (COVID-19 cluster 22), which were upregulated in both diseases, are receptors for the histocompatibility system and have important functions in antigen presentation, while the upregulated genes of clusters 6 and 32 (related to COVID-19 cluster 18) are related to intracellular transport (vesicle and organelle movement) and cell cycle regulation. Other related clusters, 1 and 3 of COVID-19 and 37 and 11 of COPD, are active in cell proliferation through ubiquitin complex formation and protein synthesis, respectively.

#### 3.4.3. IPF–COPD Connectivity

The analysis of functional proximity between IPF and COPD clusters revealed closeness between IPF clusters 10, 1, 3, 9, and 7 and COPD clusters 13, 23, 8, 4, and 3, respectively (see red boxes in Figure 6). The expression profiles of these comparisons, less IPF cluster 1 with COPD cluster 23, were inversely related (upregulated in one and downregulated in the other). For example, IPF cluster 10 and COPD cluster 1 showed the greatest homology, but these were upregulated in IPF and downregulated in COPD. Representative genes in this cluster were involved in Wnt signaling. These important genes, which were not differentially expressed in COVID-19, are involved in tissue regeneration and fibroblast proliferation. IPF cluster 1 and COPD cluster 23 are chemokine-related genes, many of which are upregulated and show functional analogy, albeit with different participating genes. COPD cluster 8 and IPF cluster 3, as in COVID-19, represent a set of genes, receptors, and immunoglobulins of immune cells that participate in regulation of the inflammatory response and are mainly downregulated in COPD and upregulated in IPF. Cluster 9 of IPF and 4 of COPD represent a cytokine profile that is clearly pro-fibrotic in the case of IPF by upregulating interleukin (IL)-11, IL-13, and IL23. Finally, IPF cluster 7 downregulated and COPD cluster 3 upregulated genes related to P450 enzymes.

## 4. Discussion

The transcriptomic analysis in this work was performed to identify the core pathophysiological mechanisms in patients with COVID-19 and to determine if they are similar to pulmonary fibroproliferative processes in IPF. We showed that patients who died of COVID-19 shared some of the molecular biological processes triggered in IPF, with the analysis of functional connectivity identifying processes related to the immune response, airway remodeling, and wound healing that indicate active fibrogenic processes. However, our mathematical approach showed a significantly large functional distance between the two diseases, which implies that both entities have more molecular divergences than could be radiologically suggested [2,31,32]. 

The highest functional association between COVID-19 and IPF was observed with IPF cluster 1, which showed the greatest closeness with COVID-19 clusters 19 and 23. However, only a few common genes from one were noted in the other. IPF cluster 1 includes a large number of chemokines (CXCLXX and CCLXX type) and their receptors (CXCRXX), as well as membrane G proteins and coupled proteins (e.g., GNG2, BDKBRB1, and BDKBRB2). In fact, the huge number of chemokines in IPF suggests a great storm involved in the recruitment of many inflammatory cell types and the perpetuation of an inflammatory state [33]. In COVID-19 clusters 19 and 23, some upregulated homologous chemokines seen in IPF cluster 1 were present, such as CXCL9, CXCL10, and CCL5, related to the regulation of immune cell migration, differentiation, and/or activation. Increased expression of other G proteins (GNG12) and coupled proteins, with downstream signaling functions related to intracellular calcium, showed a similar functional relationship with those in IPF, although differences in the isoforms could have been due to differences in the composition of the inflammatory cell infiltration [33]. Therefore, although we observed a functional analogy between the IPF and COVID-19 chemokine clusters, the differences in number of genes and type of isoforms seem to maintain a functional distance that indicates these are different fibrogenic responses. Consistent with the differences in fibrogenic responses, the observed change in the cytokine profile in IPF (cluster 9) was related to pro-fibrotic processes (e.g., increased expression of IL13, IL23, and IL11) [34], but was not observed in COVID-19. By contrast, we saw a decrease in cytokines related to the remodeling of the extracellular matrix in COVID-19 (e.g., decreased expression of IL24 and insulin-like growth factor). Patients with COVID-19 also lacked activation of a pathway that has been strongly related to IPF, such as the WNT signaling pathway in IPF (cluster 10), which has been observed to be downregulated in COPD (cluster 1) [12,35]. Both dependent and independent WNT/β-catenin pathways contribute to the cellular phenotypes that trigger and facilitate fibrosis [36,37], so the non-activation of this pathway in COVID-19 could suggest a less severe stage of pulmonary fibrosis. 

IPF cluster 3 showed functional coincidence with clusters 12 and 15 of COVID-19. This set of genes is related to receptors and immunoglobulins of B and T lymphocytes (e.g., CD3, BLK, CD79, IGHXX) involved in the dynamic regulation of the inflammatory response and the complement-mediated promotion of the humoral response to viral infection [38].

There was an interesting comparison between the main altered clusters in patients with COVID-19 and those identified in patients with COPD, a disease that is etiologically different from IPF but is also characterized by the presence of an inflammatory and vascular fibrogenic component [12]. Many common genes were downregulated, with the greatest proximity being between cluster 10 of COVID-19 and cluster 15 of COPD. These small clusters group several proteins of the ADAMTS (a disintegrin and metalloproteinase with thrombospondin motifs) family that have metalloproteinase functions, and in some members, have been related to the inhibition of endothelial proliferation through vascular endothelial growth factor sequestration [39,40]. They have attributed functions that are necessary for normal growth and structural development, so their inhibition can have negative consequences on organ development [41], as well as play a role in microvascular hemostasis [42]. Another proximal relationship was found between cluster 21 of COVID-19 and clusters 28 and 30 of COPD. The main downregulated genes in these clusters were related to mitogen-activated protein kinase (MAPK) and structural-related proteins, such as lysyl oxidase (LOX) and fibulin (FBLN). Interestingly the downregulation of both LOX and FBLN5 has been associated with remodeling of the vascular extracellular matrix (ECM), induced by the inflammatory component [43]. LOX is central to ECM maturation and seems to be crucial to preserving endothelial barrier function. Evidence suggests a role for this enzyme in atherogenesis and endothelial dysfunction, triggered by atherosclerotic risk factors and pro-inflammatory cytokines [43]. The alteration of LOX and fibulin fits well with the concept that COVID-19 patients experience endothelial dysfunction.

As was seen with IPF, cluster 19 of COVID-19 grouped several important chemokines related to upregulated and downregulated genes in cluster 23 of COPD. The roles of several CXCLXX/CXCRXX axes have been reviewed in COPD pathophysiology [44]. The CXCL9–CXCR3 axis, which was upregulated in COVID-19, is thought to be involved in the recruitment of Th1 and CD8+ lymphocytes to sites of inflammation in COPD [45], with the subsequent immune-mediated lung damage occurring through the production of perforins and granzyme B [46]. Of additional interest is the relationship of upregulated genes between clusters 2 and 18 of COVID-19 with clusters 31, 32, and 6 of COPD. Genes from clusters 18, 32, and 6 are involved in regulating cell cycle, proliferation, cell division, and apoptosis (e.g., via survivins, cyclins, and kinesins). Several of these genes have been linked to vascular remodeling and pulmonary hypertension [47,48]. Cluster 2 of COVID-19 and cluster 31 of COPD contain proteins related to lysosomal content (enzymes and proteases) that are particularly relevant in the context of pulmonary disease due to their ability to exert elastase activity, inactivate airway host defense proteins, induce ECM remodeling, and alter mucus production [49].

Taken together, our findings have some important implications that warrant consideration. Firstly, some studies have revealed that the images of organized fibrosis observed in patients with COVID-19, although similar to those that found in patients with IPF, probably suggest a persistent fibrotic lung entity other than IPF [11]. This may, therefore, result in a more favorable long-term prognosis. Interestingly, recent studies have shown that a significant proportion of patients with radiological abnormalities within 3 months after hospital discharge improve considerably at 1-year follow-up [8,9,10]. Secondly, the fact that several of the altered molecular pathways present in COPD overlap with those in COVID-19 suggests that the impact on this subpopulation could be significant. Regretfully, there is no long-term follow-up survey on lung function decline in patients with COPD who have survived to SARS-CoV-1, SARS-CoV-2, or Middle East Respiratory Syndrome to clarify this point. However, existing data indicate that patients with COPD are at increased risk of severe pneumonia and poor outcomes if they develop COVID-19 [50,51]. Furthermore, active smoking, the main cause of COPD, is a recognized risk factor for a complicated course of COVID-19 [52]. In a recent study of 110 patients admitted for COVID-19 pneumonia who did not require admission to intensive care, 47% experienced abnormalities in the DLCO without other ventilatory defects (e.g., in the FVC, FEV1, or FEV1/FVC ratio), suggesting that this was due to involvement of the alveolocapillary barrier rather than occupation of alveolar volume or airway involvement [52]. In another study in Spain, it was shown that 11% of 850 patients admitted for COVID-19 presented a mild obstructive alteration at discharge, and although some cases could have been due to previous functional alteration (66% were smokers), a direct impact of infection could not be ruled out [53]. Thus, patients with COPD who overcome COVID-19 can show long-term sequelae that have a high impact on global health. It should also be considered that patients with early COPD, or even heavy smokers with normal lung function (GOLD 0–1), could experience accelerated lung function decline if additional lung damage was caused by the infection.

This study has some technical limitations. Firstly, the microarray studies were carried out in three different laboratories with different methodologies. Therefore, to avoid undermining the analysis of similarities and differences within databases, we did not compare the levels of gene expression directly, and instead performed a meta-analysis of only DEGs. Secondly, although the medical records of COVID-19-infected patients did not report a chronic lung disease in any of them, the lack of lung function tests meant we could not determine if these patients had a prior diagnosis of pulmonary pathology. Finally, a larger number of individuals would make our conclusions more robust. However, emerging long-term studies on radiological fibrotic imaging seem to confirm those differences in the transcriptomic profile between IPF and COVID-19 patients.

## 5. Conclusions

This study was developed to identify common molecular pathways, related to fibrotic mechanisms, between COVID-19 and IPF. However, the lack of clear functional connectivity between clusters indicated that a higher proportion of patients with COVID-19 will not end up developing a fibroproliferative process similar to IPF. Instead, our findings suggest that the images of organized fibrosis observed in patients with COVID-19, although similar to those in patients with IPF, would fit with other fibrotic pneumopathies that have more favorable prognoses, such as organized pneumonia or proliferative diffuse alveolar damage. Surprisingly, there was marked functional proximity between the molecular pathways altered in COVID-19 and those altered in COPD, suggesting that COPD patients who have overcome COVID-19 could worsen their lung functional state. In addition, future research could determine if there is a real increase in the prevalence of COPD after the COVID-19 pandemic and what impact this has on the health system.

## Figures and Tables

**Table 1:** Biological processes of the Gene Ontology (GO) database.

biological process	GO:0002376	immune system process
biological process	GO:0045087	innate immune response
biological process	GO:0006954	inflammatory response
biological process	GO:0019882	antigen processing and presentation
biological process	GO:0009611	response to wounding
biological process	GO:0048771	tissue remodeling
biological process	GO:0001837	epithelial to mesenchymal transition
biological process	GO:0043043	peptide biosynthetic process
biological process	GO:0006766	vitamin metabolic process
biological process	GO:0006935	chemotaxis
biological process	GO:0001775	cell activation
biological process	GO:0008283	cell population proliferation
biological process	GO:0050900	leukocyte migration
biological process	GO:0048870	cell motility
biological process	GO:0051301	cell division
biological process	GO:0000278	mitotic cell cycle
biological process	GO:0016049	cell growth
biological process	GO:0007155	cell adhesion
biological process	GO:0007165	signal transduction
biological process	GO:0007259	receptor signaling pathway via JAK-STAT
biological process	GO:0000165	MAPK cascade
biological process	GO:0014065	phosphatidylinositol 3-kinase signaling
molecular function	GO:0003824	catalytic activity
molecular function	GO:0008009	chemokine activity
molecular function	GO:0005125	cytokine activity
molecular function	GO:0005216	ion channel activity
molecular function	GO:0019814	immunoglobulin complex
molecular function	GO:0031012	extracellular matrix
molecular function	GO:0008083	growth factor activity
molecular function	GO:0003924	GTPase activity
molecular function	GO:0016209	antioxidant activity
molecular function	GO:0006508	proteolysis

## Data Availability

The datasets generated (excel files) during and/or analyzed during the current study are not publicly available due to the size of the files but are available from the corresponding author on request.

## Supplementary Materials

The following supporting information can be downloaded at: https://www.mdpi.com/article/10.3390/life12060887/s1, Figure S1. Complete clusters for IPF; Figure S2. Complete clusters for COPD; Figure S3. Complete clusters for COVID-19.

## Abbreviations

COVID-19: coronavirus disease 2019; IPF: idiopathic pulmonary fibrosis; COPD: chronic obstructive pulmonary disease; SARS-CoV: severe acute respiratory syndrome coronavirus; DLCO: diffusing capacity of the lungs for carbon monoxide; DEGs: differentially expressed genes; HGNC: HUGO Gene Nomenclature Committee; GO: Gene Ontology; Wnt: wingless-related integration site; IL: interleukin; GNG: protein subunit gamma; BDKBRB1: bradikidin receptor B1; BDKBRB2: bradikidin receptor B2; CXCL: C-X-C motif chemokine ligand; ADAMTS: a disintegrin and metalloproteinase with thrombospondin motifs; LOX: lysyl oxidase; FBLN: fibulin; ECM: extracellular matrix; MAPK: mitogen-activated protein kinase; BLK: B lymphocyte kinase; IGHXX: immunoglobulin heavy chain XX; CD: cluster differentiation.

## References

[1] Olliaro P.L. (2021). An integrated understanding of long-term sequelae after acute COVID-19. Lancet Respir. Med..

[2] Mo X., Jian W., Su Z., Chen M., Peng H., Peng P., Lei C., Chen R., Zhong N., Li S. (2020). Abnormal pulmonary function in COVID-19 patients at time of hospital discharge. Eur. Respir. J..

[3] Sibila O., Albacar N., Perea L., Faner R., Torralba Y., Hernandez-Gonzalez F., Moisés J., Sanchez-Ruano N., Sequeira-Aymar E., Badia J.R. (2021). Lung Function sequelae in COVID-19 Patients 3 Months After Hospital Discharge. Arch. Bronconeumol..

[4] Wang Y., Dong C., Hu Y., Li C., Ren Q., Zhang X., Shi H., Zhou M. (2020). Temporal Changes of CT Findings in 90 Patients with COVID-19 Pneumonia: A Longitudinal Study. Radiology.

[5] Spagnolo P., Balestro E., Aliberti S., Cocconcelli E., Biondini D., Della Casa G., Sverzellati N., Maher T.M. (2020). Pulmonary fibrosis secondary to COVID-19: A call to arms?. Lancet Respir. Med..

[6] Zhang P., Li J., Liu H., Han N., Ju J., Kou Y., Chen L., Jiang M., Pan F., Zheng Y. (2020). Long-term bone and lung consequences associated with hospital-acquired severe acute respiratory syndrome: A 15-year follow-up from a prospective cohort study. Bone Res..

[7] Wu X., Dong D., Ma D. (2016). Thin-Section Computed Tomography Manifestations During Convalescence and Long-Term Follow-Up of Patients with Severe Acute Respiratory Syndrome (SARS). Med. Sci. Monit..

[8] Wu X., Liu X., Zhou Y., Yu H., Li R., Zhan Q., Ni F., Fang S., Lu Y., Ding X. (2021). 3-month, 6-month, 9-month, and 12-month respiratory outcomes in patients following COVID-19-related hospitalisation: A prospective study. Lancet Respir. Med..

[9] Vijayakumar B., Tonkin J., Devaraj A., Philip K.E.J., Orton C.M., Desai S.R., Shah P.L. (2022). CT Lung Abnormalities after COVID-19 at 3 Months and 1 Year after Hospital Discharge. Radiology.

[10] Besutti G., Monelli F., Schirò S., Milone F., Ottone M., Spaggiari L., Facciolongo N., Salvarani C., Croci S., Pattacini P. (2022). Follow-Up CT Patterns of Residual Lung Abnormalities in Severe COVID-19 Pneumonia Survivors: A Multicenter Retrospective Study. Tomography.

[11] Ambardar S., Hightower S., Huprikar N., Chung K., Singhal A., Collen J. (2021). Post-COVID-19 Pulmonary Fibrosis: Novel Sequelae of the Current Pandemic. J. Clin. Med..

[12] Chilosi M., Poletti V., Rossi A. (2012). The pathogenesis of COPD and IPF: Distinct horns of the same devil?. Respir. Res..

[13] Lee H., Choi H., Yang B., Lee S.-K., Park T.S., Park D.W., Moon J.-Y., Kim T.-H., Sohn J.W., Yoon H.J. (2021). Interstitial lung disease increases susceptibility to and severity of COVID-19. Eur. Respir. J..

[14] Polverino F., Kheradmand F. (2021). COVID-19, COPD, and AECOPD: Immunological, Epidemiological, and Clinical Aspects. Front. Med..

[15] Desai N., Neyaz A., Szabolcs A., Shih A.R., Chen J.H., Thapar V., Nieman L.T., Solovyov A., Mehta A., Lieb D.J. (2020). Temporal and spatial heterogeneity of host response to SARS-CoV-2 pulmonary infection. Nat. Commun..

[16] DePianto D.J., Chandriani S., Abbas A.R., Jia G., N’Diaye E.N., Caplazi P., E Kauder S., Biswas S., Karnik S.K., Ha C. (2014). Heterogeneous gene expression signatures correspond to distinct lung pathologies and biomarkers of disease severity in idiopathic pulmonary fibrosis. Thorax.

[17] Van Dyck E., Nazarov P.V., Muller A., Nicot N., Bosseler M., Pierson S., Van Moer K., Palissot V., Mascaux C., Knolle U. (2014). Bronchial airway gene expression in smokers with lung or head and neck cancer. Cancer Med..

[18] Barabási A.-L., Gulbahce N., Loscalzo J. (2010). Network medicine: A network-based approach to human disease. Nat. Rev. Genet..

[19] Jassal B., Matthews L., Viteri G., Gong C., Lorente P., Fabregat A., Sidiropoulos K., Cook J., Gillespie M., Haw R. (2020). The reactome pathway knowledgebase. Nucleic Acids Res..

[20] Von Mering C. (2003). STRING: A database of predicted functional associations between proteins. Nucleic Acids Res..

[21] Huttlin E.L., Bruckner R.J., Paulo J.A., Cannon J.R., Ting L., Baltier K., Colby G., Gebreab F., Gygi M.P., Parzen H. (2017). Architecture of the human interactome defines protein communities and disease networks. Nature.

[22] Wang Z., Zhang J. (2007). In Search of the Biological Significance of Modular Structures in Protein Networks. PLoS Comput. Biol..

[23] Enright A.J., Van Dongen S., Ouzounis C.A. (2002). An efficient algorithm for large-scale detection of protein families. Nucleic Acids Res..

[24] The Gene Ontology Consortium (2019). The Gene Ontology Resource: 20 years and still GOing strong. Nucleic Acids Res..

[25] Ogris C., Guala D., Helleday T., Sonnhammer E. (2016). A novel method for crosstalk analysis of biological networks: Improving accuracy of pathway annotation. Nucleic Acids Res..

[26] McCormack T., Frings O., Alexeyenko A., Sonnhammer E.L.L. (2013). Statistical Assessment of Crosstalk Enrichment between Gene Groups in Biological Networks. PLoS ONE.

[27] Ogris C., Helleday T., Sonnhammer E.L. (2016). PathwAX: A web server for network crosstalk based pathway annotation. Nucleic Acids Res..

[28] Ghiassian S.D., Menche J., Barabási A.-L. (2015). A DIseAse MOdule Detection (DIAMOnD) Algorithm Derived from a Systematic Analysis of Connectivity Patterns of Disease Proteins in the Human Interactome. PLoS Comput. Biol..

[29] Hennig C. (2015). fpc: Flexible Procedures for Clustering. http://cran.r-project.org/package=fpc.

[30] Galili T. (2015). dendextend: An R package for visualizing, adjusting and comparing trees of hierarchical clustering. Bioinformatics.

[31] Yu M., Liu Y., Xu D., Zhang R., Lan L., Xu H. (2020). Prediction of the Development of Pulmonary Fibrosis Using Serial Thin-Section CT and Clinical Features in Patients Discharged after Treatment for COVID-19 Pneumonia. Korean J. Radiol..

[32] Zhao Y.-M., Shang Y.-M., Song W.-B., Li Q.-Q., Xie H., Xu Q.-F., Jia J.-L., Li L.-M., Mao H.-L., Zhou X.-M. (2020). Follow-up study of the pulmonary function and related physiological characteristics of COVID-19 survivors three months after recovery. eClinicalMedicine.

[33] Desai O., Winkler J., Minasyan M., Herzog E.L. (2018). The Role of Immune and Inflammatory Cells in Idiopathic Pulmonary Fibrosis. Front. Med..

[34] She Y.X., Yu Q.Y., Tang X.X. (2021). Role of interleukins in the pathogenesis of pulmonary fibrosis. Cell Death Discov..

[35] Shi J., Li F., Luo M., Wei J., Liu X. (2017). Distinct Roles of Wnt/*β*-Catenin Signaling in the Pathogenesis of Chronic Obstructive Pulmonary Disease and Idiopathic Pulmonary Fibrosis. Mediat. Inflamm..

[36] A Baarsma H., Königshoff M. (2017). *‘WNT-er is coming’*: WNT signalling in chronic lung diseases. Thorax.

[37] Burgy O., Königshoff M. (2018). The WNT signaling pathways in wound healing and fibrosis. Matrix Biol..

[38] Nielsen C.H., Fischer E.M., Leslie R.G.Q. (2000). The role of complement in the acquired immune response. Immunology.

[39] Hsu Y.-P., Staton C.A., Cross N., Buttle D.J. (2012). Anti-angiogenic properties of ADAMTS-4 in vitro. Int. J. Exp. Pathol..

[40] Lambert J., Makin K., Akbareian S., Johnson R., Alghamdi A.A.A., Robinson S.D., Edwards D.R. (2020). ADAMTS-1 and syndecan-4 intersect in the regulation of cell migration and angiogenesis. J. Cell Sci..

[41] Hirohata S., Inagaki J., Ohtsuki T. (2017). Diverse Functions of a Disintegrin and Metalloproteinase with Thrombospondin Motif-1. YAKUGAKU ZASSHI.

[42] Katneni U.K., Alexaki A., Hunt R.C., Schiller T., DiCuccio M., Buehler P.W., Ibla J.C., Kimchi-Sarfaty C. (2020). Coagulopathy and Thrombosis as a Result of Severe COVID-19 Infection: A Microvascular Focus. Thromb. Haemost..

[43] Rodríguez C., Martínez-González J. (2019). The Role of Lysyl Oxidase Enzymes in Cardiac Function and Remodeling. Cells.

[44] Henrot P., Prevel R., Berger P., Dupin I. (2019). Chemokines in COPD: From Implication to Therapeutic Use. Int. J. Mol. Sci..

[45] Saetta M., Mariani M., Panina-Bordignon P., Turato G., Buonsanti C., Baraldo S., Bellettato C.M., Papi A., Corbetta L., Zuin R. (2002). Increased Expression of the Chemokine Receptor CXCR3 and Its Ligand CXCL10 in Peripheral Airways of Smokers with Chronic Obstructive Pulmonary Disease. Am. J. Respir. Crit. Care Med..

[46] Cosio M.G., Majo J., Cosio M.G. (2002). Inflammation of the Airways and Lung Parenchyma in COPD. Chest.

[47] McMurtry M.S., Archer S.L., Altieri D.C., Bonnet S., Haromy A., Harry G., Bonnet S., Puttagunta L., Michelakis E.D. (2005). Gene therapy targeting survivin selectively induces pulmonary vascular apoptosis and reverses pulmonary arterial hypertension. J. Clin. Investig..

[48] Weiss A., Neubauer M.C., Yerabolu D., Kojonazarov B., Schlueter B.C., Neubert L., Jonigk D., Baal N., Ruppert C., Dorfmuller P. (2019). Targeting cyclin-dependent kinases for the treatment of pulmonary arterial hypertension. Nat. Commun..

[49] Brown R., Nath S., Lora A., Samaha G., Elgamal Z., Kaiser R., Taggart C., Weldon S., Geraghty P. (2020). Cathepsin S: Investigating an old player in lung disease pathogenesis, comorbidities, and potential therapeutics. Respir. Res..

[50] A Attaway A., Zein J., Hatipoğlu U.S. (2020). SARS-CoV-2 infection in the COPD population is associated with increased healthcare utilization: An analysis of Cleveland clinic’s COVID-19 registry. eClinicalMedicine.

[51] Leung J.M., Niikura M., Yang C.W.T., Sin D.D. (2020). COVID-19 and COPD. Eur. Respir. J..

[52] Alqahtani J.S., Oyelade T., Aldhahir A.M., Alghamdi S.M., Almehmadi M., Alqahtani A.S., Quaderi S., Mandal S., Hurst J.R. (2020). Prevalence, Severity and Mortality associated with COPD and Smoking in patients with COVID-19: A Rapid Systematic Review and Meta-Analysis. PLoS ONE.

[53] Huguet E.T., Gajarte A.U., Iturriaga L.A.R., Fernandez L.S., Malanda N.M., Pascual M.I., Jorge R.Z. (2020). Alteración funcional pulmonar en el seguimiento precoz de pacientes con neumonía por COVID-19. Arch. Bronconeumol..

