# Supplementary figures and images for: Differences and Similarities between the Lung Transcriptomic Profiles of COVID-19, COPD, and IPF Patients: A Meta-Analysis Study of Pathophysiological Signaling Pathways
Source: Life (Basel). 2022 Jun 14;12(6):887. doi: 10.3390/life12060887 (PMC9227224; doi:10.3390/life12060887)

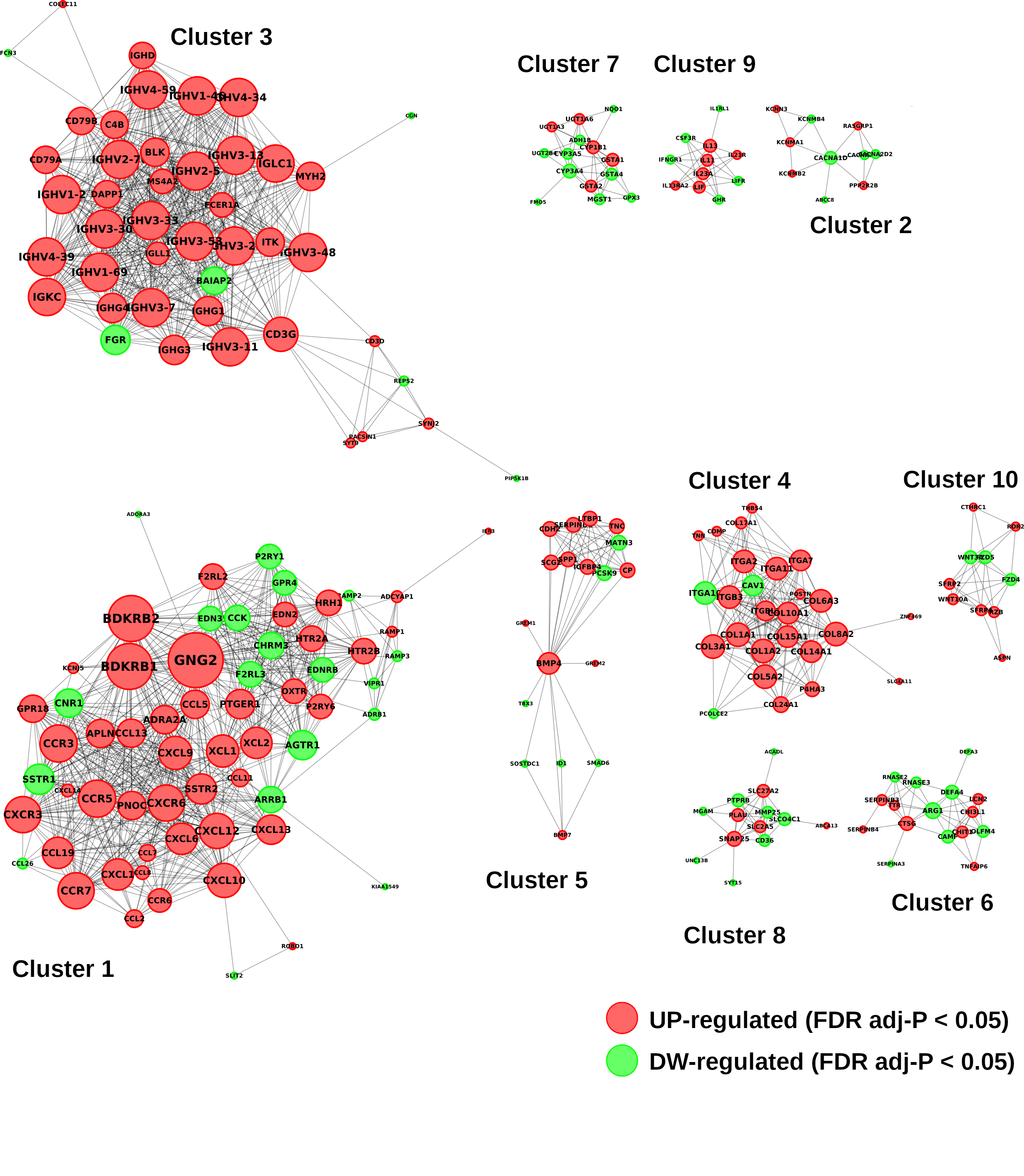

Supplement: Supplementary file 1 [file life-12-00887-s001.zip › Supplementary Figure_S1.tiff]

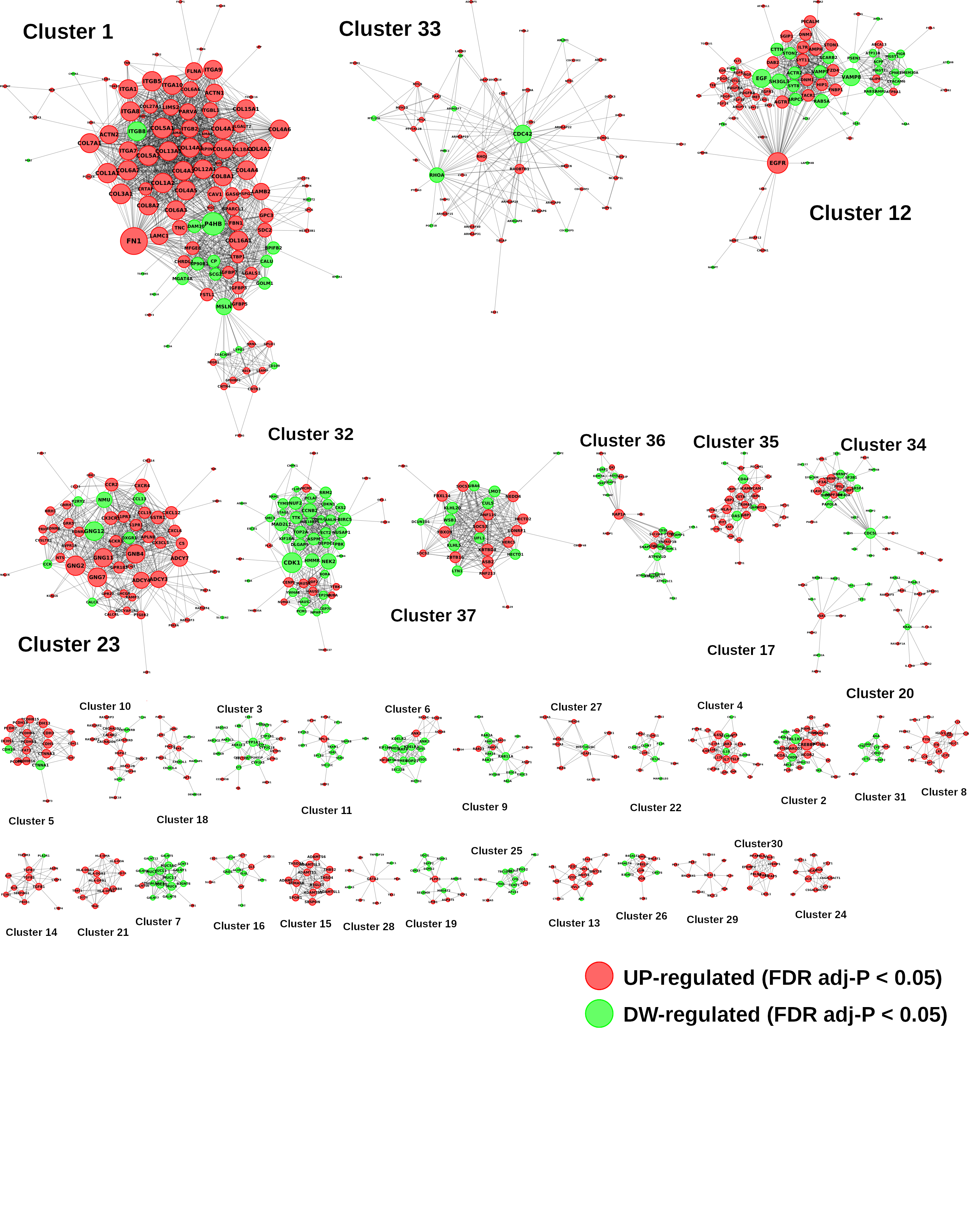

Supplement: Supplementary file 1 [file life-12-00887-s001.zip › Supplementary Figure_S2.tiff]

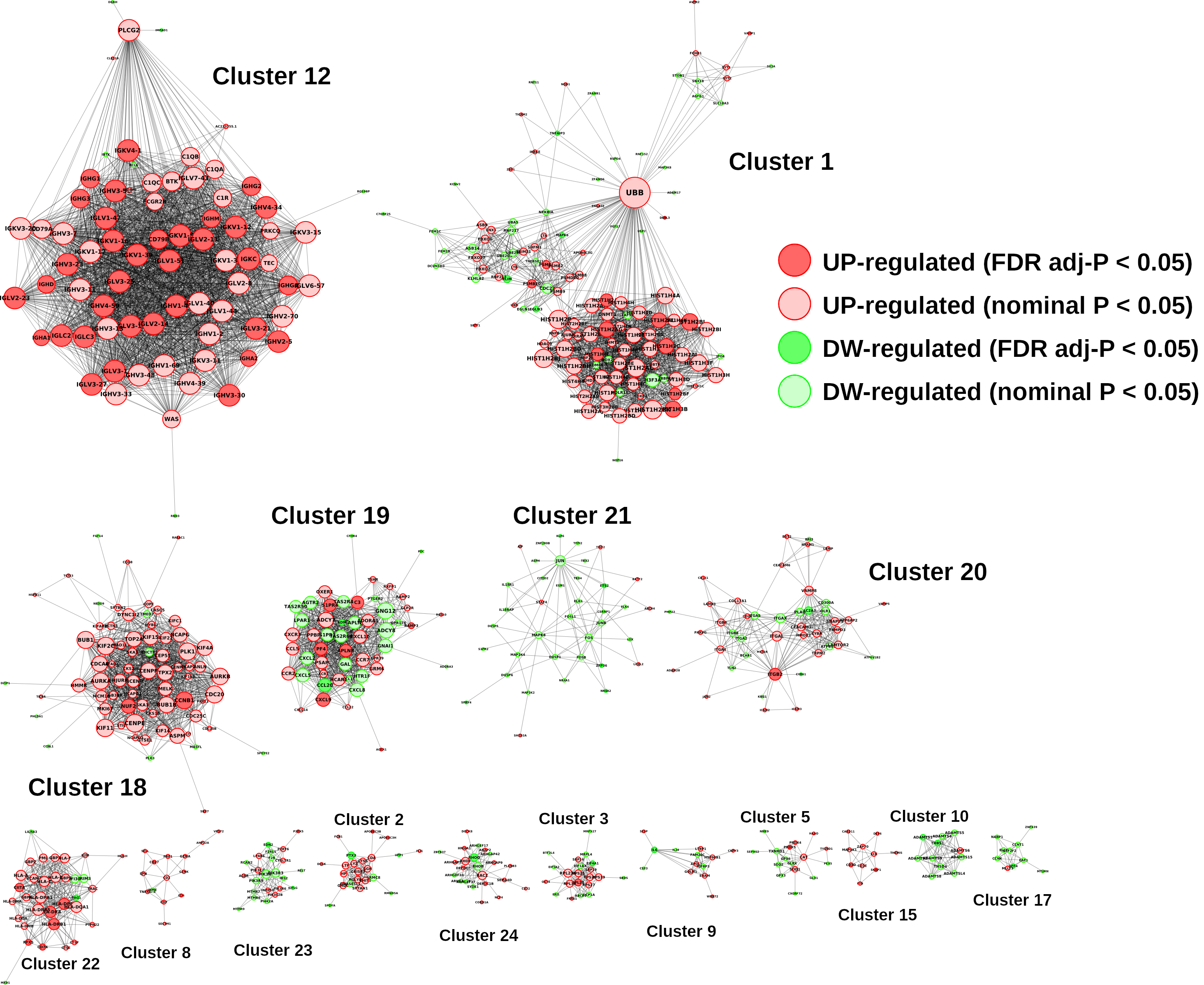

Supplement: Supplementary file 1 [file life-12-00887-s001.zip › Supplementary Figure_S3.tiff]
